# Effects of Physical Cues on Stem Cell-Derived Extracellular Vesicles toward Neuropathy Applications

**DOI:** 10.3390/biomedicines12030489

**Published:** 2024-02-22

**Authors:** Danyale Berry, Justice Ene, Aakash Nathani, Mandip Singh, Yan Li, Changchun Zeng

**Affiliations:** 1 Department of Industrial and Manufacturing Engineering, FAMU-FSU College of Engineering, Florida Agricultural and Mechanical University, Tallahassee, FL 32310, USA; danyale1.berry@famu.edu; 2 High Performance Materials Institute, FAMU-FSU College of Engineering, Florida State University, Tallahassee, FL 23210, USA; 3 Department of Chemical and Biomedical Engineering, FAMU-FSU College of Engineering, Florida State University, Tallahassee, FL 32310, USA; je17d@fsu.edu; 4 College of Pharmacy and Pharmaceutical Sciences, Florida Agricultural and Mechanical University, Tallahassee, FL 32307, USA; aakash1.nathani@famu.edu (A.N.); mandip.sachdeva@famu.edu (M.S.)

**Keywords:** peripheral neuropathy, stem cells, extracellular vesicles, electrical stimulation, neurogenesis

## Abstract

The peripheral nervous system undergoes sufficient stress when affected by diabetic conditions, chemotherapeutic drugs, and personal injury. Consequently, peripheral neuropathy arises as the most common complication, leading to debilitating symptoms that significantly alter the quality and way of life. The resulting chronic pain requires a treatment approach that does not simply mask the accompanying symptoms but provides the necessary external environment and neurotrophic factors that will effectively facilitate nerve regeneration. Under normal conditions, the peripheral nervous system self-regenerates very slowly. The rate of progression is further hindered by the development of fibrosis and scar tissue formation, which does not allow sufficient neurite outgrowth to the target site. By incorporating scaffolding supplemented with secretome derived from human mesenchymal stem cells, it is hypothesized that neurotrophic factors and cellular signaling can facilitate the optimal microenvironment for nerve reinnervation. However, conventional methods of secretory vesicle production are low yield, thus requiring improved methods to enhance paracrine secretions. This report highlights the state-of-the-art methods of neuropathy treatment as well as methods to optimize the clinical application of stem cells and derived secretory vesicles for nerve regeneration.

## 1. Introduction

Communication between the central nervous system (CNS) and the peripheral nervous system (PNS) is essential for successful operation of the human body [1,2]. This crosstalk occurs via chemical cues translated as electrical signals [1,2]. During embryonic development, localized neural crest stem cells invaginate to form the brain and the spinal cord, which expand from the neural tube [3]. Later, neural crest stem cells migrate to the dorsal region of the embryo, differentiate into a mesenchymal lineage of PNS derivatives, and give rise to efferent (motor) and afferent (sensory) neurons [3]. Efferent neurons receive signals relayed as motor function, whereas afferent neurons return sensory information translated to chemical signals by the CNS [1,2]. For efficient communication, an intricate network of nerve fibers or axons is required to transmit signals that ensure the self-regulation of bodily systems, the collection of sensory information, and the execution of motor function [1,2]. Axons within the spinal cord branch out and transmit signals from the CNS to the peripheral regions [4]. An inhibitory or excitatory electrical impulse travels through the dendrites to the cell body, then propagates down the axon to the synapse, where information is relayed to the target area [5]. The propagation of the electrical signal is due to Schwann cells (SCs) forming protective segments of myelin sheath separated by nodes of Ranvier to increase conductivity [4,6,7]. Axons within the PNS elongate throughout the trunk and the upper and lower extremities, forming the somatic and autonomic nervous system [1,4,8]. The somatic nervous system encompasses voluntary muscular functions influenced by conscious decisions [1]. The autonomic nervous system governs involuntary function including the regulation of survival instincts and functionality of the circulatory, digestive, urinary, and reproductive systems [1,9]. Short-term and long-term trauma to the PNS can be fatal, causing decreased function of autonomic systems, altered motor function, inaccurate sensory input, and delayed sensory response from the CNS. When peripheral nerves become damaged or diseased, communication with the CNS is disrupted, leading to the most common neurological disease, peripheral neuropathy (PN) [10].

PN is estimated to affect approximately 8.8% of the world population by 2040, primarily affecting older generations [11,12,13,14]. Those affected commonly experience muscular atrophy, decreased sensation, mobility, balance, and coordination [13,15]. Other associated symptoms include symmetrical pain, numbness, tingling, or burning sensations at the distal end of the upper and lower extremities [12,16]. As the PN and associated pain continue to escalate, affected people will experience increased risk of further injury due to muscular weakness, altered gait cycle, decreased joint movement and range of motion, and in the worst-case scenario, complete paralysis [13,17,18]. Depending upon the injury mechanism, the recommended treatment approach for superficial injuries encompasses noninvasive therapy. Once the nerve gap surpasses tensionless reconstruction, stem cell-based therapies can provide supportive growth factors to accelerate the natural healing process, potentially replacing conventional invasive therapeutic methods [2]. The intervention of stem cells can assist in maintaining the cellular microenvironment and supplementation of secretory vesicles compacted with regenerative growth factors necessary for reinnervation [19,20]. Thus, understanding the interaction of stem cells with an injured nerve is crucial to successfully manipulate the cellular microenvironment, avoiding unethical complications [21]. Therefore, further investigation into the mechanisms guiding the regenerative influence of stem cells and the increased production of secretory vesicles will effectively manage chronic pain and treat neuropathy [22].

## 2. Mechanisms of Peripheral Neuropathies

Understanding the diverse etiology behind PN is beneficial to developing appropriate diagnoses and courses of treatment [10]. The accurate identification of the specific condition that attributes to neuropathy is difficult due to a variety of methods used in the diagnosis and classification of origin [15]. Accounting for less than 30%, patients with idiopathic neuropathy are diagnosed without an established mechanism of injury [12,13,23]. Based on diagnosis, neuropathic symptoms are attributed to genetic or developed acquisition, axonal, or demyelinated degeneration and acute or chronic injury progression [12]. According to the method of nerve degeneration, PN can present in several forms such as distal symmetric neuropathy (DSN), sensory neuropathy (SN), and autonomic neuropathy (AN) [10,12].

DSN, also known as length-dependent neuropathy, originates in the most distal portion of the lower extremities and progresses symmetrically to more proximal regions of the body [24]. On the other hand, SN negatively affects proprioception, which reduces thermal and pain sensibility in the upper extremities and more proximal regions, eventually causing complete loss of sensation in the lower and more distal extremities [24]. Finally, AN affects the sympathetic and parasympathetic nervous systems, leading to the neurological dysfunction of one or more organ system [25]. Alongside an in-depth diagnostic workup, the various symptoms of each form of neuropathy can be utilized to identify the underlying cause [10]. To complete a diagnostic evaluation, patient history, neurological examination, assessment of symptom distribution, and further laboratory testing are required to categorize symptoms in preestablished clinical patterns [10]. However, multiple etiologies of neuropathy can be active at once, highlighting the importance of early diagnosis, optimal treatment, and preventative measures to further decrease the occurrence of PN [10]. Within this section, the most prevalent mechanisms of PN are summarized and the specific forms and symptoms of neuropathy are detailed.

### 2.1. Diabetic Peripheral Neuropathy

Diabetes is prevalent in 6.4% of the worldwide population and is estimated to impact 439 million individuals by 2030 [12,26]. Of all the diabetic patients, 30–50% suffer from diabetic peripheral neuropathy (DPN). Type 1 and type 2 diabetes results from the downregulation of insulin production and absorption, respectively, which disrupts glucose regulation [27]. High levels of glucose in the vasculature affect cellular metabolism and energy production in peripheral nerves, ultimately leading to DPN [14,26,28,29]. Typically, cellular respiration via glucose phosphorylation and glycolysis pathways provides a mechanism of transporting electrons when converting between the oxidative nicotinamide adenine dinucleotide (NAD+) and reductive nicotinamide adenine dinucleotide (NADH) to produce adenosine triphosphate (ATP) in the mitochondria [27,30,31,32]. However, excess glucose promotes an excess supply of NADH, leading to imbalanced NADH/NAD+ redox signaling [30,32].

This imbalanced environment increases reactive oxygen species (ROS), negatively affecting mitochondrial metabolism and respiration and insulin insufficiency [27,33,34,35,36]. Huang et al. investigated the correlation between hyperglycemia and mitochondrial dysfunction concerning neurodegeneration in streptozotocin (STZ)-diabetic rats [34]. As the concentration of glucose increases and insulin uptake decreases, glycolysis discontinues, resulting in ATP depletion, uncontrolled oxidative stress, the downregulation of neurotrophic factors, decreased neurite outgrowth, and the induction of PN [27,30,34,37,38]. As represented in Table 1, DPN is categorized into five major categories based on the type of nerve affected or where that effect occurs.

Prevalent in more than 80% of patients affected by DPN, length-dependent neuropathy is the most common, typically described as chronic and symmetrically distributed pain, affecting first, more minor, then larger nerves until numb [14,23,24,42,43]. Focal and multifocal diabetic neuropathies such as oculomotor dysfunction and carpal tunnel are atypical, affecting a singular or small bundle of nerves within the cranial, trunk, or limb regions [14,42,44,45,46]. AN mediates the dysfunction of the urinary, reproductive, gastrointestinal, and cardiac systems, prevalent in less than 65% of both type I and type II diabetics [14,23,47,48]. Finally, diabetic amyotrophy presents as acute anterior burning when touched, pain, and muscular weakness in the quadriceps with spontaneous improvement after months of deterioration [42]. Diabetic neuropathy affects everyone differently, causing neurodegeneration in various forms within the central and peripheral nervous systems [49]. The variation in injury distribution directs treatment toward eliminating the underlying condition and managing developing symptoms [50].

Consequently, the development of chronic diabetic sensory and autonomic neuropathy affects the individual’s overall health and finances. DPN requires therapeutic and financial support to adequately manage the emotional, social, and mental health burdens of diabetes and DPN [38,51,52]. Poor adjustment to lifestyle changes necessary to maintain glycemic and psychological control can result in blindness, kidney failure, amputations, and increased risk of anxiety and depression [14,53,54]. To assist, appointments with health care providers and medical specialists, equipment, medication, and living assistance accumulates costly societal and direct expenses [14,55]. In the United Kingdom, DPN has been estimated to cost approximately $£18 billion in direct and £25 billion in indirect societal costs by 2035 [56,57]. Individually, DPN patients spend between $9632 and $24,702 annually, depending upon the form of neuropathy and severity of their condition [14,58].

### 2.2. Chemotherapy-Induced Peripheral Neuropathy

Cancerous cells are treated with antineoplastic agents that despite optimizing patient survival, introduce life-threatening side effects that can hinder a healthy physical and psychological way of life [59,60]. Depending on the type of chemotherapy, dosage, and duration of treatment, approximately 40% of patients experience chemotherapy-induced peripheral neuropathy (CIPN) [59,60]. CIPN includes progressive length-dependent sensorimotor and autonomic neuropathies caused by prescribed neurotoxic drugs. Chemotherapy is an individualized course of treatment that works to eliminate malignant tumors but also plagues the body with chronic toxicity and compromised immunity [60,61,62]. It is required that oncologists consider pre-existing conditions and the drug’s unpredictable side effects to prevent cancer remission [63,64]. Unfortunately, the utilized drugs are not target-specific [65,66]. Both malignant and healthy cells are inhibited once exposed to the maximum tolerated dose capable of reducing uncontrolled proliferation [67,68]. Although there is a reduction in cancerous agents, the side effect of CIPN becomes more prevalent due to the type, dosage, and administration of the drugs, especially in patients with pre-existing conditions [64,69]. The drugs used to treat various types of cancer include platinum compounds, taxanes, vinca alkaloids, immunomodulators, and proteosome inhibitors, as summarized in Table 2. As a result, patients experience hair loss, bone marrow toxicity, immunosuppression, decreased appetite, and induced nausea and vomiting [65,70,71].

Chemotherapeutic agents affect fundamental cellular processes including axonal transport, mitosis, cellular movement, and the management of metabolic and oxidative stress [67,68,72,73,74]. Cellular communication is possible through the release of neurotransmitters to the presynaptic terminal. Microtubules transport the chemicals along the axon to the presynaptic terminal, which are then secreted as vesicles to receptors in the postsynaptic membrane [1,75,76]. The action potential depolarizes the cellular membrane to activate voltage-gated ion channels that release neurotransmitters via exocytosis [75,77,78]. Neurotransmitter receptors, located in the membrane of the postsynaptic neuron, receive inhibitory or excitatory chemicals via endocytosis to process sensory information and generate muscular contraction [79,80]. Platinum compounds, taxanes, and vinca alkaloids disrupt microtubule function in axonal transport as well as cellular division and homeostatic regulation [74,81,82,83]. Under oxidative stress, microtubules initiate the release of inflammatory cytokines, directly effecting signaling pathways such as the mitogen-activated protein kinase (MAPK), regulated by stress-activated C-Jun N-terminal kinase (JNK) and p38 MAPK [67,73,81,82,84,85,86]. MAPK pathways respond to external stimulus that influences cellular function such as proliferation, differentiation, and senescence [85,86,87]. The activation of JNKs are the result of MAPK phosphorylation, further influencing cellular growth, death, and survival [88]. Lower levels of p38 MAPK are closely related to the autophagy of damaged organelles, playing an active role in cellular survival by tending to homeostatic functions [84]. Chemotherapeutic agents rely on the hyperactivation of p38 MAPK to inhibit cellular growth and activate genotoxic stress-induced apoptosis by disrupting spindle assembly within mitosis [84,85,87]. Neurotoxic agents that promote microtubule dysfunction and the disruption of homeostatic cellular signaling cause CIPN since the chemical cues, proteins, and nutrients required for nerve communication are inhibited [89,90].

### 2.3. Peripheral Neuropathy via Physical Injury

**Figure 1:**
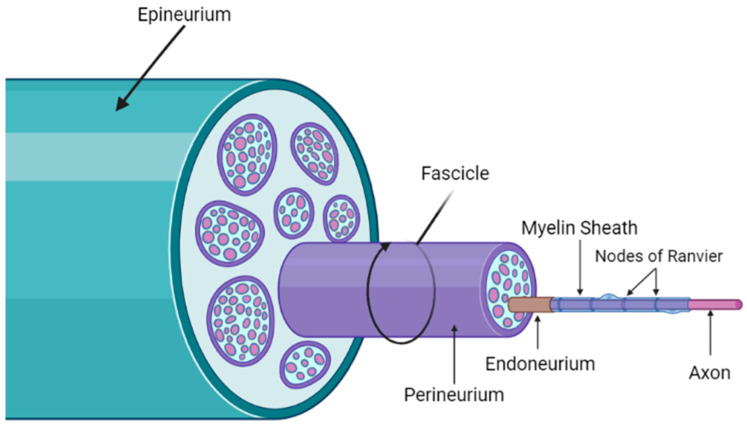
Structural representation of the nerve in PNS.

Physical injury to the nerve instantaneously alters the quality of life of those affected. Trauma to the PNS includes repetitive physical movements, the mechanical deformation of nerves, lacerations, and ischemia [91,92]. Complete recovery from such trauma depends on the severity of the injury. According to the Seddon and Sunderland classification systems, peripheral nerve injuries are divided into five categories [92,93]. To further understand how various degrees of injury are organized, the structural composition of the nerve is shown in Figure 1.

The epineurium composes the outermost layer of connective tissue, grouping together all fascicles of one peripheral nerve [94]. A bundle of nerve fibers forms a fascicle, surrounded by the perineurium, which protects the nerve by providing it with tensile strength and elasticity [95,96,97,98]. Each myelinated nerve fiber is surrounded by the endoneurium, maintaining fluidic pressure between the endoneurial space and the surrounding environment [93,96,99,100,101,102]. Beneath the endoneurium, SCs form a nutritional and protective layer of myelin sheath around the axon [101,102,103]. The axon conducts an action potential from the cell body to the nerve terminal, modulating the release of neurotransmitters [104]. Between each segment of myelin, nodes of Ranvier propagate the transmission of electrical impulses between nerves [101,102,103]. Once the myelin sheath is damaged, the rate of electrical transmission decreases, often diagnosed as a form of PN [103].

**Figure 2:**
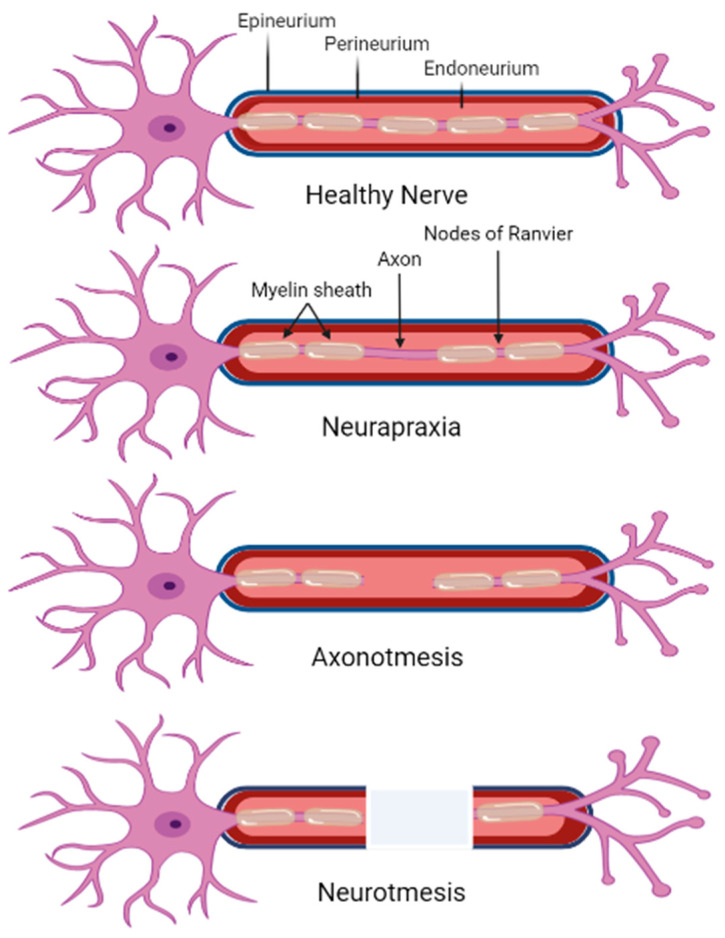
Classification of peripheral nerve injuries.

Peripheral nerve injuries are classified into three primary categories by Seddon and further defined by severity by Sunderland, as shown in Figure 2. Seddon’s method of classification developed from observed nerve injuries during World War I, focusing on conduction blocks, loss of axon continuity, and complete nerve transection [105,106]. However, Sunderland’s focused on the histological structure of each injury [105]. Seddon first defines neuropraxia as the mildest form of nerve injury caused by blockage or compression [93,107]. Neuropraxias are equivalent to Sunderland’s description of first-degree injuries [92,98,99]. First-degree injuries primarily block the transmission of electrical impulses without permitting further injury to the axon. Seddon defines axonotmesis, which is the severity of the axon, endoneurium, and perineurium, with little effect on the epineurium [92,99,106,108,109]. Sutherland further describes axonotmesis as second-and third-degree injuries [105,110]. Within second-degree injuries, the axon experiences discontinuity, but the endoneurium and perineurium are still intact [2,98,105,111]. Third-degree injuries damage the axon and endoneurium; however, the perineurium is complete [105,106,110]. This process is attributable to the SC release of cellular signals and the recruitment of macrophages to engulf axonal and myelin debris and begin regeneration [112,113,114,115]. Seddon then defines neurotmesis, which is the loss of anatomical continuity within the three layers surrounding the axon [93,109]. In this case, the event of axonal regeneration without intervention is rare [93]. Sunderland describes neurotmesis as fourth- and fifth-degree injuries. Within a fourth-degree injury, the axon, endoneurium, and perineurium are discontinuous, but the epineurium, the outermost layer, is intact [2,110]. Without the guidance of the endoneurium and perineurium, the regenerating axons return unorganized and are constricted by the development of fibrosis and scar tissue blocks. Finally, fifth-degree injuries describe the complete severance of the nerve, requiring medical interventions to treat [92,105,106,108,110].

Beyond third-degree injuries, nerve regeneration becomes increasingly difficult due to impaired axon recovery. Minimal interventions are necessary for complete axon reinnervation with returned functionality to treat first- and second-degree injuries [93,109]. When the injury is substantial enough to damage the perineurium or introduce a nerve gap, surgical and noninvasive treatment methods are practiced to manage symptoms and promote nerve regeneration [108].

### 2.4. Pathophysiology of Axonal Injury

To maintain neurons distant from the cell body to the synaptic terminal, dynein and kinesin motors actively transport intracellular cargo along axonal microtubules within the cytoskeleton [116,117]. The transport components include secretory membrane vesicles, essential organelles (mitochondria, lysosomes, and lipids), and messenger ribonucleic acid (mRNA) to maintain cellular polarity and migration [118,119,120]. Following axonotmesis, Wallerian degeneration (WD) occurs at the distal end, causing the axon to self-degenerate in preparation for reinnervation [2,7,121,122]. However, with the discovery of the Wallerian degeneration slow (Wlds) gene mutation in mice, axonal degradation is recognized as an active process [123,124].

Molecular homeostasis of the cellular environment is maintained by the neuroprotective properties of nicotinamide mononucleotide adenylyltransferase (NMNAT) and the NAD+ regulation of gene expression and metabolic and signaling processes [125,126,127]. NAD+ serves as an essential catalyst for enzymes that regulate energy metabolism, the management of ROS, and overall health of the cell [126,127]. The biosynthesis of NAD+ occurs in three primary pathways with varied precursors requiring several steps for synthesis [128]. The Preiss–Handler pathway converts nicotinic acid (NA) into NAD+ in three steps [126,128,129]. The de novo pathway synthesizes tryptophan (Trp) in 10 steps [126,128,129]. Finally, the salvage pathway can occur in a 2-step process with either nicotinamide (NAM) or nicotinamide riboside (NR) [126,128,129,130].

The disrupted production of NAD+ is primarily associated with decreased physiological and metabolic functions associated with age and the progression of neurodegenerative diseases, presenting a similar inflammatory response when an axonal injury occurs [131,132]. Demonstrated by metabolic flux analysis, Sasaki et al. concluded that axon fragmentation is induced when sterile alpha and toll/interleukin-1 receptor motif-containing 1 (SARM1) activates an increase in NAD+ synthesis until depletion due to the discontinuation of NMNAT2 [133]. Once NAD+ is consumed by SRAM1 via MAPK signaling, the decreased supply of ATP further expedites WD [134]. However, the overexpression of NMNAT or nicotinamide mononucleotide (NMN) de-amidase halts SARM1 activation, proving neuroprotective abilities following injury [133].

To facilitate the microenvironment for regeneration, subsets of glial cells such as SCs, astrocytes, microglia, and neural progenitor cells promote migration accuracy [135]. Via axo–glial interaction, c-Jun and notch transcription factors within the MAPK kinase extracellular signaling regulation pathway (MEK/ERK) promote the upregulation of transforming growth factor-beta 1 (TGF-β1) and growth factor secretion in repairing SCs (rSCs) [20,136]. As the rSCs develop uninterrupted alignment, the growth of Büngner bands are directionally influenced for successful reinnervation [2,20,137]. This guidance is provided by the influence of macrophages, fibroblasts, and SCs toward self-renewal [2]. Macrophages secrete cytokines of two different phenotypes, pro-inflammatory (M1) or anti-inflammatory (M2), further divided into four subsets (M2a, M2b, M2c, M2d) [138,139]. The fluctuating levels of M1 and M2 macrophages influence the migration, proliferation, and secretion patterns of SCs at the site of injury [138]. Until the fourth day post injury, M1 macrophages engulf fragmented axons and myelin debris during WD [2,138,140,141]. Following the first phase of regeneration, M1 macrophages are polarized to subsets of M2 macrophages to further promote immunoregulation, tissue repair and remodeling, and long-distance axonal growth [138,139]. As treatment progresses, hypoxic conditions promote M2d secretion of vascular endothelial growth factor (VEGF) to extend blood vessels across gaps between nerve segments [138,140,142,143]. The regrown vasculature serves as guidance for bands of Büngner to the target endoneurial tubes [19,138,140,142]. However, the probability of connection without growth misdirection is slim [144]. Uncontrolled branching of growing axons leads to misdirection from the target site [7]. The accumulation of dense scar tissue and fibrosis resulting from fibroblast proliferation flares up inflammation, distracts axonal regeneration, and endoneurial tube reinnervation [2].

## 3. Approaches to Peripheral Nerve Injury Treatment

The PNS is capable of self-regenerating at a rate of ~1 mm/day [2,7,20,92,98]. Grade 1 and 2 injuries will heal within a few months without assistance [2,7,98]. However, injuries of a high caliber that traumatize the axon and surrounding connective tissue require medical interventions to promote remyelination and active healing [93]. Prior to medical interventions, the primary goal of diagnosis and treatment is to mitigate the underlying mechanism of injury [138,139]. DPN and CIPN progressively alter the external environment encompassing the nerve. To halt neuropathy progression and ensure treatment to the nerve is not reversed, the elimination of toxic medication and nutritional deficiency is the goal. Within personal injuries, the nerve trauma is more instantaneous, leading to a noninvasive therapeutic approach or surgical procedure that will guide regeneration [2].

Noninvasive strategies that promote remyelination include over-the-skin electrical stimulation, steroid hormone therapy, and pharmacological agents. Once a nerve experiences interrupted stimulation to the neuromuscular junction, the release of neurotransmitters for muscular contraction is halted [145]. Therefore, voluntary and involuntary muscular contractions are impaired, increasing neuropathic symptoms [146]. To alleviate pain and improve neuromuscular activity, the PNS is exposed to transcutaneous electrical nerve stimulation (TENS) [147,148]. Electrodes are placed over the skin to stimulate the nerve and induce muscle contraction [145]. The stimulation is modified between high (>80 Hz) or low (<10 Hz) frequency, duration, and intensity depending upon the severity of neuropathic pain (NP) [147,149,150]. As a result, the clinical application of TENS increases blood circulation and axonal transport, decreases inflammation, and reinnervates muscle and nerve fibers [150,151]. Although TENS can alleviate NP, research supporting the influence of TENS in nerve regeneration is limited [152]. In a separate study, TENS has also been observed to reduce the axon count, disorganize cellular arrangement within the tissue, and negatively affect the remaining nerves at the injury site [2]. Externally applied stimuli hinder morphological development in nerve regeneration but positively effect sensory-motor function [152]. Hormonal steroids influence the regulation of physiological functions within the CNS and PNS [153]. These steroids modulate pain sensitivity while providing neuroprotection and the maintenance of SC myelination when PNS nerve injury occurs [153,154,155]. The utilization of estradiol in neuropathy management treatment directs functional improvement and the regeneration of injured peripheral nerves [156]. In a study by Calabrese et al., animals experiencing pain caused by DPN were treated with testosterone metabolites [157]. DPN induces the expression of toll-like receptor member 4 (TLR4), which increases the production of inflammatory cytokines causing NP [157,158]. Once treated with testosterone metabolites, 3α-diol and dihydrotestosterone (DHT), pro anti-inflammatory cytokines were counteracted, increasing the analgesic properties [157].

The pharmacological treatment of NP is focused on the management and relief of symptoms [159,160]. Commonly, the combination of anti-depressants, anti-convulsant, opioids, and natural products work to reduce the perception of pain resulting from neural hyperexcitation [159,160]. First-line drugs such as tricyclic antidepressants, lidocaine, phenytoin, and capsaicin inhibit the transduction of voltage-gated channels, ligand-gated channels, G protein-coupled receptors (GPCRs), and gamma-aminobutyric acid (GABA) receptors [161]. Natural components including omega-3, curcumin, berberine, lycopene, and naringin possess anti-inflammatory properties by inhibiting the expression of injury-induced chemokines and cytokines [161]. Second- and third-line drugs such as opioids also reduce NP, however, adverse side effects and a high dosage required for effective treatment discourages usage [161]. Once treated with natural or synthetically derived drugs, patients commonly experience a placebo effect or a euphoric relief from pain, without treating the original mechanism [159,160]. Alternatively, topical agents such as tacrolimus (FK506), hyaluronic acid (HA), melatonin, lidocaine, and vitamin B12 actively support the alleviation of NP [147]. Tarcolimus (FK506) counteracts neurotoxicity by increasing the expression of growth-associated protein 43 (GAP-43), known for neuronal plasticity and regeneration [162,163]. HA is naturally found in the extracellular matrix composition, stimulating cluster of differentiation 44 (CD44) expression following a traumatic nerve injury [164,165]. HA can provide a suitable environment for nerve regeneration and recovery when topically administered [165]. Lidocaine targets the mechanism of neurotransmitter release, inhibiting the generation of an action potential required for nerve signal conductance [147]. Finally, vitamin B12 promotes myelination and upregulates gene transcription factors for nerve regeneration and pain management [166]. However, this approach to treatment is passive, lacking specificity to the various types of neuropathies and accompanying symptoms [147,159].

Invasive techniques used to treat severe nerve injuries include nerve graft, allograft, nerve transfer, and conduits. The U.S. Food and Drug Administration (FDA) has approved conduits that are primarily constructed with collagen or hyaluronic acid hydrogels, or synthesized with poly-glycolic acid (PGA), polycaprolactone (PCL), and polyvinyl acetate (PVA), effectively re-establishing functionality (Table 3) [167]. In vitro and rodent-based trials (in vivo) have occurred with a combination of natural and synthetic materials, exploring manufacturing techniques for nerve injury treatment. Standard fabrication methods include dip coating, solvent casting, freeze-drying, micro-patterning, and additive manufacturing [168]. The dip coating, solvent casting, and freeze-drying methods produce conduits with varying sizes and connectivity of pores, decreasing the transfer of nutrients and metabolic waste [168]. With electrospinning and micropatterning, the fiber network resembles the extracellular matrix (ECM) and allows for the strategic alignment of growing axons; however, low reproducibility is a disadvantage [168]. Finally, additive manufacturing is highly reproducible and can control specific morphological features depending on the printing method and material used [168].

Protein-based hydrogels and synthetic conduits are engineered with varied concentrations of crosslinking agents to influence the biophysical and biochemical cues that promote cellular proliferation, the secretion of ECM components from seeded SCs, and the organization of regrowing axons [169,178,179]. Adjusting the crosslinking agent allows for the effective treatment of severe injuries of a significant distance and large diameter [169]. Biophysical properties such as the porosity, stiffness, degradation, and biochemical communication between protein binding sites and proliferating cells ensure nutritional support as regenerating axons close the nerve gap [169,179,180,181].

## 4. Neurotrophic Support in Neuropathy Treatment

With natural or biomaterials acting alone, conduits lack the mechanical and structural properties necessary to support axon regeneration. However, combining biodegradable polymers with biological proteins produces a biocomposite conduit capable of regulating the biochemical cues and growth factors necessary to support neurite outgrowth without disrupting the surrounding connective tissue [117,178,182]. Growth factors are released from the distal and proximal nerve stumps to generate axoplasmic fluid, forming a neomatrix of fibrin [167,180]. Nerve injuries with significant gaps and are large in diameter have limited neurotrophic support; therefore, additional nutrients are required to see nerve reinnervation to completion [180]. Recent studies support the seeding of stem cells and growth factors within biocomposite conduits to enhance the neomatrix between nerve stumps [108,183,184]. To further increase the probability of axon regeneration, neurotrophic factors such as nerve growth factor (NGF), brain-derived neurotrophic factor (BDNF), glial cell-derived neurotrophic factor (GDNF), and VEGF are released from SCs and localized along the conduit [2,168]. Neurotrophic factors within conduits assist in promoting SC migration, neuronal survival, and axon regeneration [168,173].

### 4.1. Stem Cell Differentiation

Stem cells are classified into two categories: embryonic (totipotent and pluripotent) and nonembryonic (multipotent, oligopotent, and unipotent), based on where they are derived [185]. Self-renewing pluripotent stem cells express transcription factors for the blastula formation that generate the three germ layers: ectoderm, mesoderm, and endotherm [186,187,188,189]. The ectoderm gives rise to the nervous system, the mesoderm gives rise to connective and muscular tissue, and the endoderm gives rise to organ systems throughout the body [190]. Multipotent stem cells, commonly derived from bone marrow, adipose tissue, or dental pulp, experience differentiation into several cell types within one designated germ line [186,187,188,191]. Once within a specific lineage, differentiation is flexible [185]. Due to increased plasticity, self-renewal, and proliferative properties, totipotent, pluripotent, and multipotent stem cells are most advantageous in tissue engineering [188]. However, there are limitations associated, primarily moral objections when harvesting embryonic stem cells and uncontrolled teratoma formation and immunorejection, once clinically applied [187,188,192].

To harvest, tissue rich in stem cells such as the umbilical cord or placenta, bone marrow, adipose tissue, and peripheral blood is first collected then filtered [188,193]. However, neonatal-derived stem cells from the umbilical cord and placenta are considered unethical, leading to the preferred use of mesenchymal stem cells (MSCs) and induced pluripotent stem cells (iPSCs) harvested from connective tissue within the body [188]. Connective tissue is preferred due to the accessibility, cost effectiveness, and abundance of MSCs, however, the harvesting technique influences the survival and yield of the cells collected [194]. Compared to liposuction, syringe aspiration is the most effective because trauma to the donor site is minimized, and the viability of the cells is maintained [194].

MSCs give rise to different mesenchymal lineages within the mesoderm based on the specific stimuli and signaling required for differentiation [193,195,196]. MSCs are derived from bone marrow and adipose tissue [193,195,197,198]. Cellular components of bone marrow and adipose tissue include sympathetic neurons, SCs, macrophages, regulatory T cells, neutrophils, fibroblasts, pericytes, and endothelial cells [195,197]. The positive markers of CD90, CD73, CD105, and negative markers of CD45, CD34, CD14, CD11b, CD19, and human leukocyte antigen (HLA-DR) effectively distinguish MSCs from other cellular components in the bone marrow (BM) and adipose tissue [193,194,198,199,200,201,202]. Depending upon the source, whether bone marrow or adipose tissue, the markers present slightly differ [194]. To influence differentiation, MSCs are exposed to specific chemical cocktails that upregulate Wnt signaling for osteogenic, chondrogenic, and cardiogenic differentiation [196,197]. Wnt signaling is highly influential in stem cell division, proliferation, migration, and fate determination to increase neurite outgrowth [5,203]. The differentiation of MSCs to a true neuronal lineage is arguable since neurons and connective tissue reside in a different lineage [190]. To encourage an altered lineage, the activation of Wnt signaling promotes the expression of neuronal and glial cell markers in MSCs after exposure to signals that will influence transdifferentiation [196,198]. The induction medium is supplemented with fibroblast growth factor (FGF), Sonic Hedgehog protein (SHH), retinoic acid (RA), and BDNF over 18 days [198]. Reverse transcription polymerase chain reaction (RT-PCR) is used to confirm the gene expression of neural phenotypes [198]. Immunocytochemistry visually confirmed the morphological changes of neurite extension typically exhibited by neural stem cells [198]. Within this study conducted by Urrutia et al., RT-PCR and immunocytochemistry identified the expression of neuroepithelial stem cell protein (NESTIN), β-tubulin III, synaptophysin, neurofilament light polypeptide (NEFL), neurofilament medium polypeptide (NEFM), dopaminergic neuron marker (NURR1), calcium-binding protein B (S100B), and neurotrophin-3 (NT-3) [198]. Comparing the multiple sources human MSCs were isolated from, neuronal markers were considerably more expressed in adipose-derived mesenchymal stem cells (ASCs) than in BM-MSCs [198].

Identical to embryonic stem cells (ESCs), iPSCs are developed from reprogrammed somatic cells or human fibroblasts by introducing growth factors [193]. In 2007, Takahashi et al. reconditioned human fibroblasts to human iPSCs by utilizing a transcription factor cocktail including octamer transcription factor 3 and 4 (OCT-3/4), sex-determining region Y-box 2 (SOX2), c-Myc gene, and Kruppel-like factor 4 (KLF4), engineered initially by Yamanaka for mice fibroblasts [204,205,206]. This discovery eliminates the need to transfer the nucleus from somatic cells [206]. However, using c-Myc leads to the death of embryonic stem cells [206]. Alternatively, Yu et al. demonstrated the successful reprogramming of human MSCs to iPSCs using OCT4, SOX2, NANOG gene, and Lin28 gene [206]. The addition of NANOG and LIN28 proved to increase the survival rate and recovery of reprogrammed cells [206]. To verify similarities between iPSCs and ESCs, RT-PCR and Western blot analysis identified comparable gene expressions and undifferentiated cell-surface markers such as OCT3/4, SOX2, NANOG, FGF4, reduced expression 1 (REX1), and growth and differentiation factor 3 (GDF3) [205,207]. Immunocytochemistry also showed consistent morphology and proliferation between the embryonic and induced pluripotent cell lines [205,207]. IPSCs can differentiate into mature neural progenitor cells and astrocytes once introduced to a neural induction medium for 21 days [208]. Kang et al. mapped the morphological, genetic expression, and electrophysiological profile changes endured during iPSCs to neuron differentiation [208]. The morphology of differentiating iPSCs detailed increased dendrites and the lengthening of axons following growth cone development throughout 15 days [208]. Immunostaining confirmed the positive gene expression of mature neurons including NESTIN, paired box 6 (PAX6), SOX2, class III beta-tubulin (Tuj1), glial fibrillary acidic protein (GFAP), synapsin 1, and tyrosine hydroxylase (TH) [208]. The genes identified also influence the upregulation of signaling pathways that regulate stem cell proliferation such as MAPK, ligand–receptor interaction, and Wnt pathways [208]. The electrophysiological profile characterization confirmed the synapse’s successful formation by recording excitatory postsynaptic currents [208]. Once the cellular membrane is depolarized, the calcium (Ca^2+^) current, decreased membrane resistance, and increased membrane capacitance are recorded, confirming signal conductance for effective neural communication [208].

Based on the specific conditions the MSCs are transplanted to, complete neural differentiation is not achieved, however, the phenotypic properties of glial cells are adopted [209]. Once injected, MSCs promote angiogenesis, anti-inflammation, and neuroprotection during the regeneration process through secretome expression by which cells exchange communicative signals [209]. Clinically, compared to MSC injections, the intravenous application of paracrine secretions has gained popularity due to the ability to modulate injury symptoms and facilitate functional recovery [209]. As a cell-free therapeutic method, secretory treatment voids the instability experienced with MSC differentiation and the safety risks associated with stem cell transplantation [210,211].

### 4.2. The Application of Stem Cells in Neuropathy Treatment

Stem cell treatment of PN-induced chronic pain provides assistive interaction with the damaged cells by inhibiting apoptosis and enhancing cellular survival during regeneration [181,212]. The primary mechanism of pain is attributed to the activation of the Janus kinase 2/signal transducer and activator of transcription 3 (JAK2/STAT3) pathway, p38-MAPK pathway, and Notch signaling once peripheral damage occurs [213]. In response to injury, nerve hyperexcitation from an immune-mediated response and continuous infiltration of proinflammatory cytokines contribute to demyelination and neuronal death [181,212,213]. By releasing anti-inflammatory, angiogenic, and nutritional neurotrophic factors such as BDNF, NT-3, FGF, and VEGF, stem cells strongly regulate the body’s natural immunoresponse when peripheral nerve damage occurs [181]. Transplantation of BM-MSCs has been proven to upregulate the expression of anti-inflammatory M2 macrophages, downregulate inflammatory M1 macrophages, and influence the MAPK signaling pathway toward the native SC response to injury [181].

Preclinical trials have primarily focused on the successful delivery and retention of stem cells in neurodegenerative diseases such as Parkinson’s disease, Huntington’s disease, and ischemic stroke [214]. Traditionally, MSCs have been introduced systemically via intracerebral, intravenous, arterial, and nasal infusion to activate neurogenesis in diseases affecting the CNS [212,213]. Stem cells can cross the blood–brain barrier (BBB), allowing effective migration toward damaged brain tissue [215]. The intracerebral application of MSCs in ischemic stroke has proven to reduce inflammation, inhibit further destruction of the BBB, and promote neurogenesis [215]. However, the limitations of this method compromise the success of treatment due to cell clusters trapped in the respiratory and circulatory system [212,215]. Alternatively, the intravenous and intraarterial application is safer but less effective as many cells do not cross the BBB and develop into blood clots or occlusions that lead to further damage [215]. Intranasal administration allows for the successful migration of stem cells via the olfactory system with MSC detection in brain tissue [215]. Proving their success in neurodegenerative diseases affecting the CNS provides gateway access to pain modulation and neuropathy treatment within the PNS [213].

MSC transplantation to the PNS improves neuropathic symptoms by inhibiting destructive mechanisms while maintaining nerve function and axonal regeneration [213]. Various studies, outlined in Table 4, support MSC mediation of oxidative stress, ROS formation, neural inflammation, and apoptosis through the secretion of neurotrophic factors [213].

Yu et al. investigated the effect of multiple intravenous infusions of ASC on systemic inflammation and the long-term complications brought on by type 2 diabetes [218]. This study aimed to demonstrate the long-term therapeutic potential of ASCs in pain management and interruption of injury progression [218]. Diabetic rats were treated with ASC infusions once a week for 24 weeks. Blood glucose levels gradually decreased to normal levels throughout treatment after each MSC infusion [218]. Insulin sensitivity increased due to the restoration of islet b cells, which is necessary for a proper pancreatic negative response to glucose [218]. MSC treatment also alleviated inflammation due to an increased expression of M2 macrophage phenotypes, effectively combating the development of fibrosis, which negatively affects other essential bodily systems [218]. Similarly, Xiang et al. concluded that treating MSCs in diabetic rats reduced the expression of proinflammatory interleukin-1b (IL-1b), IL-6, and tumor necrosis factor (TNF-α) and reduced the M1 macrophage secretion of TGF-β within the kidneys [216]. Xiang et al. also observed the secretion of anti-inflammatory and anti-fibrotic factors that effectively improved renal function and inhibited the harmful progression of DPN [216]. Within CIPN, the administration of MSCs reversed the pain in mice exposed to chemotherapeutic medications known to negatively affect mitochondrial function and increase oxidative stress [220,221]. After 24 days of MSC treatment, mitochondrial respiration was restored, attributed to the increased MSC and M2 macrophage expression of IL-10 signaling [221]. The limitations of the systemic administration of MSCs are still an issue, requiring an abundant amount of MSCs in the hope that they stay viable, successfully differentiate, and accurately treat the desired target site [220,224].

**Figure 3:**
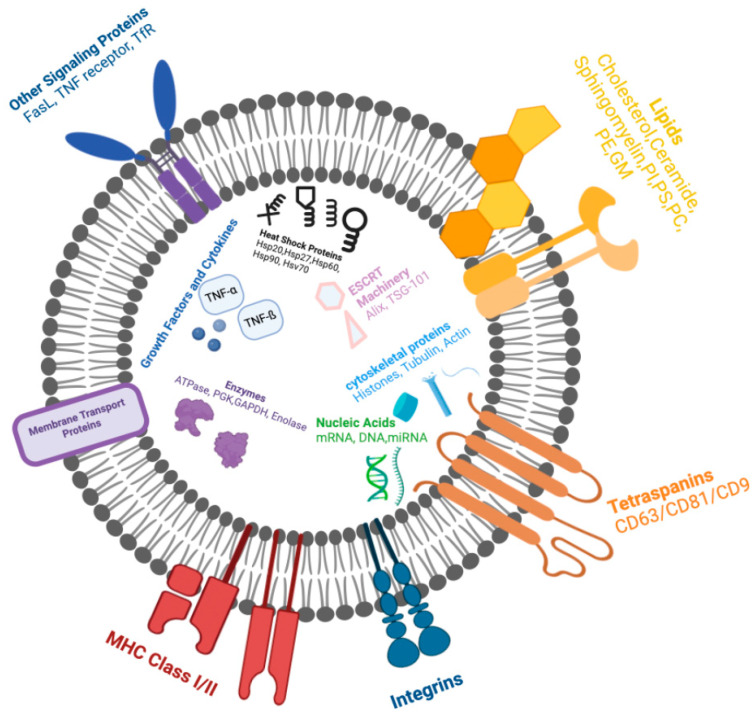
Components of EV cargo. Exosomes are composed of a multitude of proteins, molecules, growth factors, cytokines, lipids, and nucleic acids that influence the exosome structure, cargo organization, secretion, and signaling in multiple biological processes.

As previously stated, the intravenous transplantation of MSCs for nerve regeneration increases the risk of complications that limit treatment capabilities. MSCs undoubtedly promote healing, however, the retention of neural differentiation and capability of effective treatment is not guaranteed [225]. The decreased survival rate requiring multiple injection and the risk of vascular obstruction contributing to stroke encourages research into a more optimal therapeutic approach [225]. Alternatively, the secretome produced from MSCs has been explored due to the production of extracellular vesicles [225]. Extracellular vesicles (EVs) are secreted organelles from the parent cell, compacted with cargo that promotes regenerative function, induces angiogenesis, and regulates cellular communication [224]. EVs range in size and function including apoptotic bodies (>1000 nm), microvesicles (100–1000 nm), and exosomes (50–150 nm) [226,227]. The variety in size requires differential centrifugation to isolate a pure sample [224,228]. The cargo of EVs is potent with proteins, lipids, mRNA, and microRNA (miRNA), which promotes an enhanced regenerative potential such as the type of cell they are derived from (Figure 3). EVs derived from a specific lineage of stem cells replicate mechanisms of intracellular communication and signaling mediation as the parent cell to encourage regenerative properties [227,228]. For example, EVs derived from differentiated neural MSCs have been shown to stimulate angiogenesis, neurite outgrowth, and regeneration and inhibit inflammation, oxidative stress, and apoptosis [224,229].

### 4.3. Potential of MSCs Secretome in Nerve Regeneration

Naturally, exosomes within the PNS regulate synaptic activity via neurotransmitters, modulate intracellular and cell-to-cell communication, and facilitate the exchange of biological information to maintain homeostatic conditions [228,230,231]. The primary mode of communication is through the transportation and selective delivery of mRNA, miRNA, and proteins from the donor to the recipient cell. The EV transfer of mRNA facilitates the paracrine exchange of genetic information [224]. MiRNAs are crucial in stimulating gene expression and facilitating cellular proliferation, differentiation, migration, and apoptosis [224]. The delivery of proteins is essential for managing tissue regeneration and providing a mechanism for the EV surface to interact with cellular receptors for targeted delivery [224]. The primary methods of EV uptake include phagocytosis, receptor-mediated endocytosis, and direct fusion with the cellular membrane [227,232]. However, efficient EV consumption depends on the biophysical and mechanical properties that allow the proper interaction for tissue absorption [227]. The size, elasticity, stiffness, and Young’s modulus of EVs differs per source of the parent cell [227]. Modification of the EV surface increases migration through the ECM and enhances the attraction of specific surface proteins to cellular receptors for more effective delivery [227].

#### 4.3.1. EV Biogenesis and Transport

**Figure 4:**
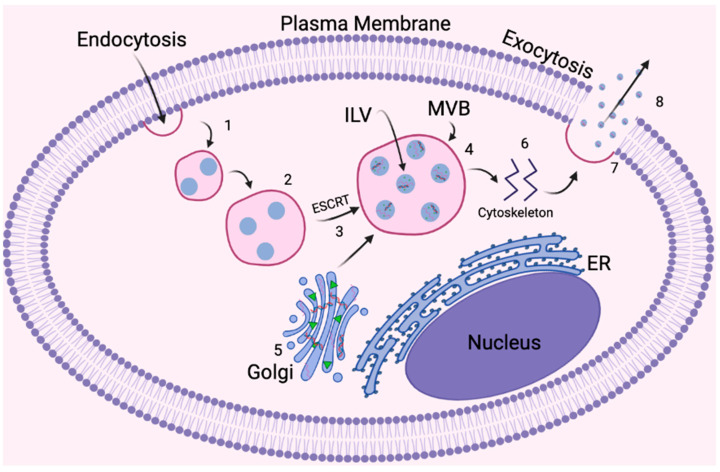
Biogenesis of exosomes. (1) Internalized cargo from the cellular membrane via endocytosis is sorted into (2) early endosomes. (3) ESCRT, tetraspanins, and lipids guide early endosomes through late endosome/MVB maturation, (4) which is concentrated with ILVs. (5) The Golgi apparatus then supplements ILVs with nucleic acids, RNAs, proteins, and MHC II molecules. (6) MVBs are then transported to the plasma membrane via the cytoskeletal and microtubule network. (7) During the transportation process, Rab GTPases guide the docking and fusion of MVB with the plasma membrane. (8) ILVs are secreted as exosomes via exocytosis.

The biogenesis of EVs is attributed to the endosomal sorting complex required for transport (ESCRT) mechanism complex, which guides endosomes through the early and late stages of development before exocytosis (Figure 4) [233]. Subunits of ESCRT promote cargo organization and internalization during intraluminal vesicle (ILV) formation, dictated by the expression of seven primary proteins: tumor susceptibility gene 101 (TSG101), Alix, chromatin modified protein 4C (CHMP4C), vascular protein sorting-associated (VPS) protein 4B, vacuolar protein sorting-associated protein (VTA1), hepatocyte growth factor-regulated tyrosine kinase substrate (Hrs), and signal transducing adaptor molecule (STAM1) [233,234,235]. Once organized within late-endosome development, the Golgi apparatus then supplies ILVs with major histocompatibility complex (MHC) class I and II molecules, growth factor receptors, and RNAs, encompassed by multivesicular bodies (MVBs) [233,236]. The MVBs are then transported to the cellular membrane, guided by cytoskeleton, microtubules, and Ras-associated binding guanosine triphosphates (Rab GTPases), then secreted as exosomes via exocytosis [233,234]. Rab GTPases are crucial during the transport of MVBs to the cellular membrane, specifically Rab27a and Rab27b, during MVB docking and intracellular trafficking [233]. The mechanism by which the cells receive cargo includes ligand–receptor interaction, binding to target receptors on the cellular membrane, membrane fusion, and complete exosome internalization via endocytosis [227,232,233,236]. EVs contain surface markers on the phospholipid bilayer membrane that are attracted to specific sites for targeted delivery [224,226,227,229].

Within neuropathy treatment, incorporating EVs enhances the repair environment with neurotrophic factors such as GDNF (SC recruitment), insulin-like growth factor 1 (IGF-1), TNF-α, and TGF to promote the regeneration of damaged tissue and improve functional recovery [180,237]. The primary source of EVs is derived from BM-MSC and ASCs due to easy access, nonimmunogenic response when transplanted, and the phenotypic alteration of immune-mediated cells responding to injury [237,238,239]. Due to the therapeutic capabilities of BM-MSCs and ASCs, derived exosomes from each respective stem cell are favorable in drug delivery and the treatment of neuropathy, neurodegenerative diseases, and cancer [237].

#### 4.3.2. Targeted Transplantation of EVs in Neuropathy Treatment

Within neuropathic treatment, the transplantation of exosomes derived from MSCs directly targeted to the injury site will activate signaling pathways that promote angiogenesis, immune response regulation, and the management of the extracellular environment [240,241]. The mentioned effects, which are also highlighted in Table 5, are attributed to the cargo within exosomes. Once EVs are applied to damaged cells, the cargo uptake leads to the downregulation of key factors that negatively affect the quality and health of the cells within the injury site. For example, Song et al. isolated EVs from healthy cortical neurons containing miR-NA-181-3p, which have been shown to suppress neuroinflammation via targeting the CXCL1 gene in astrocytes [242]. Alternatively, EV cargo isolation from damaged PC12 cells containing miRNA-21-5p, known to cause chronic neuro-inflammation, upregulated the expression of proinflammatory factors following EV uptake in BV2 cells [243]. The RNA within EV cargo is critical to their therapeutic effect. MiRNA-133b, a regulator of tyrosine hydroxylase production and dopamine transporter, is the best understood MSC-EV mediated treatment regarding cerebral injury [244]. Researchers have transferred MSC-EVs to injured neurons, successfully promoting neural plasticity and neurite outgrowth due to its role in post-transcriptional gene regulation and neuroprotective upregulation [244,245]. However, a more directed approach to treatment will accelerate the healing capabilities associated with EVs. The uptake of EVs continues to be a challenging obstacle to overcome. When injected intravenously, EVs primarily accumulate in organs of the reticuloendothelial system such as the liver or spleen [246]. A further understanding of site-specific EV uptake is needed, specifically toward neuropathy treatment.

Once applied, the uptake of EVs may occur via five different mechanisms including clathrin-dependent and independent endocytosis, caveolin-mediated invagination, lipid raft-mediated endocytosis, phagocytosis, and macropinocytosis [233]. Clathrin-mediated endocytosis occurs by forming a clathrin-coated vesicle due to deformation in the plasma membrane cytoskeleton [233,254]. The inward budding vesicle is separated from the membrane by dynamin-2 and then further developed through the endocytic pathway [233,254]. Similarly, caveolae-mediated invagination creates a membrane specifically concentrated with glycoproteins and cholesterol, recognized as caveolae [233,254]. Lipid-raft mediated endocytosis is an invagination process that is enriched in cholesterol, sphingolipids, and glycosylphosphatidylinositol (GPI)-anchored proteins, promoting the formation of early endosome [233]. Phagocytosis internalizes EVs into a large vacuole through the rearrangement of the membrane cytoskeleton, identified by the phagocytic marker, lysosomal-associated membrane protein 1 (LAMP-1) [233,254,255]. Alternatively, macropinocytosis promotes the rearrangement of the cytoskeleton into lamellipodia to engulf nonspecific EVs into lysosomes [233,255]. Each method of internalization can co-exist and co-occur [233]. To specifically direct EVs toward damaged nerves for neuropathic treatment, utilizing EVs derived from the parent cell known to be directly involved in nerve injury treatment may influence the targeting capabilities [256]. Common EV markers identified for neuronal regeneration, EV biogenesis, and uptake by neuronal cells include CD81, CD9, and CD63 [257,258]. Further research on the surface protein and chemical dependence is needed to understand which mechanisms govern EV uptake toward neuropathy treatment.

SCs play an influential role in the maintenance of the PNS. The plasticity of SCs allows the transdifferentiation from mature myelinating SCs to immature SCs, then rSCs that initiate the neuroinflammatory response causing NP [259]. Following the dissipation of injured nerve fragments, rSCs supply neurotrophic factors for axonal regeneration, alter the phenotype of immune-responsive M1 macrophages to anti-inflammatory M2 macrophages, and guide Büngner bands to the target site [239]. Due to their crucial role in nerve regeneration, recent studies have explored the application of rSC-derived exosomes in neural regeneration and neuropathic treatment [239]. Further investigation strives to identify the miRNA composition with rSC-derived exosomes directly influencing axonal growth [260]. El-Derany et al. treated CIPN with exosomes derived from BM-MSCs, successfully identifying exosomal miRNAs (miR-21-5p, miR-125b-5p, miR-199a-3p, miR-24-3p, and let-7a-5p) secreted to the damaged nerves [248]. López-Leal et al. demonstrated increased neurite outgrowth once rSC exosomes transferred miRNA21 to damaged tissue, directly activating the expression of c-Jun, SOX2, and the modified regulation of regenerative molecules via the phosphatidylinositol 3-kinase (P13K)/protein kinase B (AKT) signaling pathway [239,261,262]. C-Jun expression activates the repair mechanism of SCs, and SOX2 activates the immune response while inhibiting myelinating factors of rSCs [261]. The PI3K/AKT pathway is active in cellular proliferation, cellular survival, cell cycle progression, cellular plasticity, glucose metabolism, and protein synthesis [262]. Targeting the PI3K/AKT pathway has been explored as a viable option to regulate diabetics and diseases affecting the nervous system [262]. For example, Li et al. successfully alleviated NP induced by chemotherapeutic agents through the supplementation of resveratrol via the PI3K/AKT signaling pathway [263]. As a result, mitochondrial dysfunction improved due to the reduction in oxidative stress and successfully alleviated NP symptoms [263]. Concerning SC function, the PI3K/AKT pathway, which regulates the tuberous sclerosis complex (TSC), activates the mechanistic target of rapamycin complex 1 (mTORC1), playing an important role in the myelination of axons and mRNA protein translation for cellular metabolism, growth, and proliferation [264,265,266,267,268]. Along with the Erk1/2 signaling pathway, this pathway is also influential in SC development and transdifferentiation in response to pathological conditions [267,268].

Of the three types of SCs, the expression of rSCs and derived exosomes is most beneficial in the axonal regeneration and management of NP [261]. However, the utilization of rSC exosomes is limited regarding the number of EVs produced, the loading efficiency of desired cargo, and retention once applied clinically [224,239]. The process of EV isolation has not been optimized to generate large enough quantities to support complete regeneration [224,237]. Additionally, nonfunctional components within EV cargo hinder effective treatment, requiring an abundance of EVs at the injury site [224,239]. Finally, the retention of rSC phenotype expression is unstable [239,269]. Once the injury gap length and time required for reinnervation become extensive, the secretion of neurotrophic factors fades as the cell-to-axon biochemical cascade no longer encourages a regenerative microenvironment [269,270].

### 4.4. Methods to Increase EV Production

The clinical application of EVs requires mass production and an optimal isolation protocol to ensure that sustainable quantities are obtained [211]. Traditional two-dimensional (2D) cultures produce a low yield of EVs and batch-to-batch variability between cellular passages [211]. A 2D culture does not actively represent the native environment in which cell-to-cell and cell-to-matrix interactions naturally occur within the ECM [271]. To mimic accurate cellular behavior, three-dimensional (3D) culture conditions allow cells to form aggregates for the increased production of EVs [272]. Furthermore, 3D aggregates enhanced by dynamic cellular agitation have been proven to allow for the large-scale production of EVs with an enhanced expression of therapeutic cargo [211,272,273,274]. Through shear stress, the expression of ESCRT-independent/dependent biogenesis markers significantly increased alongside EV production within the PBS Vertical-Wheel Bioreactor compared to the 2D culture [273]. As a result, the EVs isolated from the 3D culture expressed upregulated therapeutic miRNA secretion consistent with angiogenesis, wound healing, and neuroprotection [273]. Jeske et al. compared the effect of shear stress within the PBS Vertical-Wheel Bioreactor to 2D static culture, investigating the secretion and cargo profile of hMSC-derived EVs [273]. Western blot analysis presented an upregulation in exosomal markers HRS, syntenin-1, CD81, and CD63 in the bioreactor groups compared to the 2D group [273]. Finally, the miRNA cargo within the 3D bioreactor groups, compared to the 2D groups, showed an upregulation in EVs that would effectively promote wound healing such as miR-10, 19a, 19b, 21, 30b, 92a, 126, and 132 [273]. Therefore, culture expansion and increased EV production rely on improving culture conditions, enhancing the external environment, and stimulating signaling pathways that influence cellular secretions [211,271]. Alternatively to the PBS Vertical-Wheel Bioreactor, the spinner flask and rotating wall bioreactors mildly support scaling up for clinical application; however, various limitations discourage utilization. For example, a horizontal impeller is used to agitate the media in spinner flask bioreactors. However, this application of shear stress results in turbulent flows and nonhomogeneous shear zones within the reactor, generating aggregates of different quality [275,276].

Alongside scaling up EV production, the ability to store generated EVs effectively while ensuring a nonsignificant drop in quality is also a limiting factor in the clinical utilization of EVs. EVs are typically stored in PBS or media at 4 °C or −80 °C, as suggested by the International Society for Extracellular Vesicles [277]. However, in 2018, these suggestions were redacted due to the impact of EV preservation and long-term storage on the stability, concentration, and overall function [278]. These storage methods have been shown to significantly decrease EV yield within seven days of storage [279]. The buffer selection, temperature, and storage techniques greatly influence the EV shelf life to optimize current storage techniques.

Regarding a storage buffer, Kawai-Harada et al. developed an EV storage buffer consisting of trehalose and BSA-supplemented PBS-HEPES buffer [279]. This buffer showed better cargo protection than the standard storage techniques without sacrificing any loss in targeting ability, size, or EV morphology [279]. While a promising alternative to standard storage methods, the study on the effects of the EV storage buffer is limited to 7 days. A clinically applicable method would need long-term storage of months to years. This EV storage buffer has yet to be tested on EVs derived from stem cells, as Kawai-Harada et al. utilized HEK293T cell-derived EVs, and it is unlikely that the necessary cargo for neuropathic treatment is stored within these EVs. Therefore, the effects the storage buffer could have on stem cell-derived EV cargo quality are unknown. Another study by Görgens et al. addressed EV storage conditions for up to two years, concluding that buffers composed of trehalose and human albumin showed significant improvement in EV preservation for samples stored at −80 °C [280]. While MSC-derived EVs were studied within this research, the data are inconclusive compared to those of the HEK293T cells utilized. Particle concentration saw a significant improvement after storage, regardless of temperature. Cellular uptake and cargo quantification were not investigated with these EVs. Another limitation, regardless of the storage solution, is that multiple freeze–thaw cycles have been shown to induce membrane disruption and re-micellization, with effects being significant after as few as two cycles [281,282]. Other avenues such as lyophilization and temperature dependency have also been explored with varying levels of success and conflicting results [281,283,284]. These alternative methods share the same limitation of short-term analysis, with no studies testing for the effects on EVs over extended periods. For EVs to be clinically ready, the enormous hurdles of successful scale and proper long-term storage protocols must be addressed to ensure the quality and repeatability of EVs between stocks.

The influence of the external environment on cellular gene expression directly alters the protein synthesis and behavior of the cell [285]. Specifically regarding ESCRT-dependent and -independent biogenesis markers, the upregulation of HRS, TSG101, Alix, sphingomyelin phosphodiesterase 2 (SMPD2), SMPD3, melanocyte including transcription factor (MITF), STAM1, and GTPases Rab27a and Rab27b expression effectively increases the exosome yield [234,273]. ESCRT-dependent exosome biogenesis markers, HRS, TSG101, and Alix, promote endosomal budding, selective cargo sorting, and MBV formation toward exosome release [235]. The ESCRT-independent biogenesis markers include the vital role of lipids and tetraspanins such as CD9, CD63, CD37, CD81, CD82, and CD53 during cargo sorting and exosome budding [235]. Ceramide is a lipid that is essential in the formation of ILVs, generated by the enzyme neutral sphingomyelinase 2 (nSMase2) [234,235]. As one of the primary mechanisms of exosome formation, upregulating nSMase2 expression alongside increased metabolic activity effectively promotes ceramide function within the ESCRT-independent pathway [234]. Once formed, GTPases Rab27a and Rab27b promote the transport and binding of MVBs for release [233,235]. The influence of external stimulus toward increased EV production has been accurately identified in the upregulation of essential exosome biogenesis markers. Wang et al. demonstrated the upregulation of MSC-derived exosomes without increasing the cellular volume [234]. The introduction of norepinephrine, fenoterol, N-methyldopamine, and forskolin to the MSC culture increased exosome production 3-fold [234]. The additional molecules effectively enhanced the nSMase2 promotion of ceramide expression as well as Rab27a and Rab27b, correlating the increased production of exosomes to an abundance of cargo [234,235]. Similarly, the microcarrier-based expansion of hMSCs within 3D PBS Vertical-Wheel Bioreactors compared to base 2D cultures effectively increased the exosome yield 2.5-fold, an upregulation of EV biogenesis markers, and the enhanced expression of neuroprotective microRNA [273]. Aside from environmental cues, direct stimulation of the signaling pathways that control the endolysosomal pathway charged with EV production may produce the largest yield [271].

#### Electrical Stimulation Promoting Transdifferentiation and EV Production

Electrical stimulation (ES) is a form of neuromodulation commonly used to stimulate damaged nerve fibers that induce chronic pain in spinal cord injuries and peripheral neuropathy [286]. The neuronal response to ES is induced neuroplasticity, which alters the synaptic release of neurotransmitters, cellular behavior, and overall response to injury [286]. Similarly, the cellular response to an electrical field promotes cellular signaling to initiate hMSC transdifferentiation toward a neural lineage and increases exosome production for therapeutic applications, evident in Table 6 [287,288]. Neural-like differentiation, neurite outgrowth, and increased exosome production are attributed to low-level ES combined with growth factors and mechanical cues from the external microenvironment [212,288,289].

ES catalyzes the role of neurotransmitters and receptors in cellular signaling, subsequently increasing the production of EVs [288]. Limited understanding of the relationship between ES and cellular signaling further promotes the investigation of ES on the natural SC response to peripheral nerve injury [290]. Enhanced neural excitation directly alters voltage-gated ion kinetics across the cellular membrane and the synaptic release of neurotransmitters that influence pain modulation and cellular functionality [286]. Hu et al. electrically stimulated dorsal root ganglion (DRG) cells with 100 mV/mm, which effectively increased cellular proliferation as well as the production of glutamate [291]. Glutamate is an excitatory neurotransmitter that mediates the peripheral nerve communication, cellular signaling, and SC secretion of exosomes [290]. Once ES is applied, excess glutamate binds with ionotropic glutamate receptors, causing an influx in Ca^2+^ [290,291]. As a result, Hu et al. highlighted the direct correlation between an upregulation in Ca^2+^ ion concentration and the increased secretion of EVs [288,291]. Similarly, Zhang et al. exposed cardiac-MSCs (C-MSCs) to low-level ES from 2 to 72 h. There was a significant increase in the nSMase2 protein levels, crucial to EV biogenesis and release [292]. Compared to C-MSCs (control), C-MSCs (ES) produced a 38% increase in EV particles/mL concentration and diameter [292]. Alternatively, with high voltage and exposure time, the hyperactive rate of nerve action potential proved to be damaging to the cell [291]. A complete understanding of ES on the cell has yet to be fully understood; however, ES has been confirmed to alter cellular energy metabolism, morphology, phenotype, and Ca^2+^ expression [291,292]. The optimization of ES parameters is necessary to ensure cellular proliferation and the secretion of glutamate and EVs without causing cellular damage or the ability to produce quality EVs. Fukuta et al. isolated EVs from electrically stimulated B16F1 and 3T3 Swiss Albino cultures that effectively increased the particle quantity without compromising the exosome quality [288]. Compared to cultures unexposed to ES, Western blot analysis reflected an insignificant difference in the expression of EV markers CD9, HSP70, and CD81 [288]. The low-level ES permits a Ca^2+^ influx to activate exosome biogenesis and Rho GTPase involvement in the mechanism of cellular exocytosis [288].

The effect of ES can be further enhanced by materials known to possess the ideal biophysical and conductive properties for enhanced EV secretion and MSC neural differentiation. For example, graphene is commonly used in scaffold manufacturing, implant devices, and substrates for cellular differentiation [256,293,294]. Despite graphene’s benefits, complex material production places its viability into question. Guo et al. utilized a reduced graphene oxide (rGO) microfiber scaffold to enhance electrical stimulation toward neural differentiation. Further modifications incorporated poly(3,4-ethylenedioxythiophene) (PEDOT), a biocompatible conductive polymer, within the rGO microfibers due to negatively charged carboxylic acid groups. This composite material is bioactive due to rGO and is highly conductive due to the PEDOT, with no significant effect on mechanical properties [212]. Compared to rGO microfibers, rGO–PEDOT hybrid microfibers maintained 99% cellular viability, and increased MSC adhesion and proliferation over five days of ES exposure. Finally, immunostaining and quantitative PCR (qPCR) results expressed Tuj1 and GFAP markers toward neural differentiation [212]. This is attributed to the enhanced electrical–cellular interface and the mechanical and topographical features that influence MSC morphology and gene expression [212].

ES has been shown to enhance the MSC microenvironment for differentiation. To mimic these physiological conditions in a closed system, Naskar et al. fabricated lab-on-a-chip microfluidic devices. They utilized polymethylmethacrylate (PMMA) as the material because it is noncytotoxic, biocompatible, and autoclavable for sterilization [287]. Conduction within the closed microenvironment is possible with pressure sensitive adhesive (PSA) tape and two stationary electrodes. This design allowed for a uniform electric field to stimulate the entire cell population simultaneously. ES was shown to strengthen the differentiation of C2C12 cells to neural-like cells due to the electrophysiological analysis of Ca^2+^ depolarization.

Electroconductive substrates have also been developed and implemented for localized ES during cell proliferation and differentiation. Many conductive polymers were investigated due to their potential as substrates for biological and medical applications, one of the most promising polymers being polyaniline (PANI). It was previously shown to be an excellent matrix that supports cardiac myoblast and nerve cell proliferation and differentiation [295,296,297]. Thrivikramn et al. attempted to understand the behavior of hMSCs grown on PANI films with tunable conductivity combined with ES. Results showed that the intermittent delivery of low-level ES (100 mV/cm) at 24-h intervals created distinct morphological changes, enhanced cytoskeletal elongation, and the expression of early neural markers such as NESTIN and beta-tubulin III, providing further evidence of the benefit of ES on both neural differentiation and cell proliferation [289].

**Table 6:** Electrical stimulation to promote transdifferentiation or increased exosome production for peripheral nerve injury treatment.

Title of Study	Cell Culture	ES	ES Duration	ES Method	Reference
Intermittent electrical stimuli for guidance of human mesenchymal stem cell lineage commitment towards neural-like cells on electroconductive substrates.	MSCs	DC; 1 mV–2 V	10 min/day, 3 days	Parallel stainless-steel electrodes PANI film	[289]
Neurogenesis-on-Chip: Electric field modulated transdifferentiation of human mesenchymal stem cell and mouse muscle precursor cell coculture.	hMSCs Murine myoblast	DC ~8 ± 0.06 mV/mm	20 h/day for 9 days	Microfluidic device; graphene oxide (GO) microfiber	[287]
Effectiveness of electrical stimulation on nerve regeneration after crush injury: Comparison between invasive and non-invasive stimulation.	Sciatic nerve crush injury	25 Hz, 1–3 mA, 0.1 ms pulse width	30 min/day 5 times/week for 6 weeks	Implanted wireless cuff electrodes	[298]
Low level electricity increases the secretion of extracellular vesicles from cultured cells.	Murine melanoma cell line, B16F1	0.34 mA/cm^2^	60 min Immediate EV isolation	Two Ag–AgCl electrodes with 2.5 cm^2^ surface areas	[288]
The frequency-dependent effect of electrical fields on the mobility of intracellular vesicles in astrocytes.	Rat astrocytes	5 mV/mm; 2 Hz	5 min of constant voltage; 0.1 nms pulse 600 total pulses	Stimulus isolator A365 with 1 KΩ resistor	[299]
Electrical stimulation increases the secretion of cardioprotective extracellular vesicles from cardiac mesenchymal stem cells.	Cardiac MSC	1.5 V/1.8 cm	2–72 h; 1.5 V/1.8 cm voltage, 0.5 Hz frequency, pulse width at 5 ms	Cultured-cell pacer system (IonOptix)	[292]

The molecular mechanism of cellular differentiation and increased EV production following ES exposure is inconclusive [299]. However, various studies have concluded on the increased mobility of secreted vesicles that transport vesicular cargo, neurotransmitters, neuromodulators, hormones, and peptides [299]. Ang et al. concluded that the effect of ES may not directly alter the EV, but the external factors that depict cytoskeleton and motor protein functionality surrounding the vesicles [299]. ES generates an action potential that increases the expression of Ca^2+^, neural marker proteins, cellular signaling pathways, and exosomal paracrine communication, effectively promoting enhanced cargo produced by MSC-derived EVs [287,300].

Naskar et al. applied a direct current (DC) of low-level ES of ~8 ± 0.06 mV/mm for 20 h/day for nine days to a hMSC and murine myoblast coculture within a microfluidic device [287]. The microfluidic device mimicked native biochemical cues and the directional orientation of the ECM to promote neural differentiation [287]. As a result, the appropriate microenvironment and ES profile successfully promoted the hMSC expression of NESTIN, Tuj1, and MAP2 and intracellular calcium-signaling, signifying neuronal synaptic activity [287]. The condition media also facilitated exosome mRNA protein translation from differentiated neural hMSCs to the myoblasts, exhibiting neural-like phenotypes and morphology [287]. DC stimulation at a low frequency has repeatedly demonstrated successful neural differentiation, increased exosome production, and enhanced axon regeneration [298]. Enhancement of the external environment further amplifies the method by which ES promotes nerve regeneration [212,289,300]. Biocompatible, electroconductive materials such as carbon, graphene, and PANI, enhanced with electrodes, are strategically engineered to mimic the ECM and induce cellular signals congruent with neural differentiation. However, the long-term integration of various biocompatible materials with the host tissue for additional manipulation of the external cellular environment requires further investigation. The studies incorporating biocompatible and electroconductive materials were conducted in vitro in controlled environments. However, Leng et al. successfully utilized carbon nanotube bucky paper in vivo to transplant human retinal pigment epithelium cells within the subretinal space of rats [301]. This study explicitly emphasized that minimal material manipulation is suitable for short-term host interaction. However, functionalization of the material surface is required to extend material capabilities beyond the retina [301].

Depending on the cell type and expected outcome, the parameters for electrical stimulation can range in frequency, direction, magnitude, and current. Therefore, optimization is challenging. However, the primary comparison between low- and high-level frequency and an alternating current (AC), DC, and pulsed current contributes toward an optimized protocol per cell type. An AC flows bidirectionally, causing the charge’s magnitude to periodically reverse [300]. A DC produces a consistent and directional charge, effectively guiding the cellular migration toward the anode or cathode [300].

Similarly, a PC, which can be a direct or alternating current, produces a unidirectional or bidirectional current, allowing a dynamic range of electrical frequency, strength, and duration [300,302]. Cellular directional migration during ES stimulation, otherwise known as electrotaxis or galvanotaxis, is influenced by the polarity of the activated intracellular signaling pathways and Golgi apparatus [300,302,303]. However, this phenomenon is cell-type dependent, with MSCs and iPSCs directed toward the anode and neural stem cell (NSC) migration toward the cathode [302,303,304]. To investigate cellular viability concerning ES duration and current, ASCs stimulated with direct and pulsed currents within a custom agar-salt electrotaxis chamber were exposed to 1200 μA for 3, 6, and 9 h [302]. There was a direct correlation between increased DC exposure and decreased cellular viability. However, the exact duration of pulsed current ES revealed minimal cell death while actively maintaining directional migration toward the anode through Golgi polarization [302].

Regarding frequency, the cellular response to low-level ES includes increased EV production, the upregulation of neural phenotype markers, and extended neurite outgrowth [288,289,300]. This is ultimately beneficial for neuropathic and nerve injury treatment. Alternatively, the high-level frequency that approaches the voltage capacity of the cellular membrane, especially for an extended duration, contributes toward decreased cellular proliferation, viability, and membrane integrity [300,302]. The method and parameters by which the ES was applied differed for each experiment. Each ES chamber was custom-built, thus decreasing the ability to reproduce results quickly. Although the ES parameters were different, favorable results were consistently produced when applying low-frequency levels.

## 5. Discussion

Damage to the PNS results from physical injury or demyelinating mechanisms that severely alter the microenvironment encompassing the nerves. The current state-of-the-art treatment methods for neuropathic injury do not effectively treat nerve degeneration but instead mask the associated chronic pain. Treating physical damage by nerve graft remains the golden standard; however, donor site morbidity diminishes its success. The transition to artificial nerve grafts utilizing biocompatible and biodegradable materials is a favorable alternative. However, the probability of complete nerve regeneration becomes less than likely as the nerve gap surpasses three centimeters, thus extending the time required for complete regeneration.

The three stages of SC differentiation mediate the pathophysiology of injury response. Once fragmented cellular debris is cleared, rSCs and distal and proximal nerve stumps supplement neurotrophic factors that initiate a cascade of biochemical cues that facilitate the remyelination of damaged nerves and accurately guide regrowing axons to the target site [180]. The signaling pathways most influential in nerve regeneration include PI3K/AKT/mTORC1, MAPK, Notch, Wnt, and JAK2/STAT3. Each pathway influences the cellular response to external stress, determining the cellular fate and the natural immunoresponse to injury. Working together, nerve regeneration is possible; however, large peripheral nerves with significant nerve gaps require additional neurotrophic support [180]. Gapped nerves require more time for neurite outgrowth, decreasing the survivability of rSCs. To overcome this limitation, supplying the nerve conduit with additional factors will maintain the regenerative microenvironment surrounding regrowing nerves. The application of MSCs expressing neural phenotypes and rSCs within the biocomposite conduit will actively modulate the surrounding area. Additionally, rSC-derived exosomes, compacted with cytokines, anti-inflammatory factors, and miRNA, will supply an optimal combination of neurotrophic factors necessary to accelerate the regeneration process.

To further promote the regenerative properties provided by the coculture of neural-like MSC, rSCs, and rSC-derived exosomes, modifications to the external environment enhance the production of rSC exosomes, increasing the probability of clinical applications. Low levels of direct electrical stimulation activate the Rho GTPase and PKC signaling pathways to increase EV production [288]. Within a lab setting, the in vitro application of electrical stimulation requires a device to supply voltage and conductive electrodes. However, the previously described schematic could be more realistic and convenient to the patient within clinical applications. Guo et al. developed a self-generating device powered by triboelectric charging [212]. Incorporating conductive, biocompatible, and biodegradable materials such as carbon nanotubes, enhanced by a triboelectric effect, is an effective method of maintaining a regenerative environment.

## 6. Conclusions

The PNS is more susceptible to damage than the CNS, which is protected by the skull and spinal column. Due to the associated symptoms, individuals affected by peripheral nerve injuries have trouble navigating through everyday life, preventing effective communication between the CNS and PNS. These symptoms are influenced by mechanisms such as the compression or severance of nerves, classified as Grade I–Grade IV injuries. Once a nerve is severed, the muscular function and sensory information that the nerve innervates are disconnected. The treatment options for various nerve injuries successfully restore function; however, the associated disadvantages discourage long-term use. Fortunately, peripheral nerves can slowly self-regenerate, encouraging the utilization of biocomposite conduits to guide and facilitate axon reinnervation. In conjunction with a biocompatible material, seeding neural-like MSCs, rSCs, and rSC-derived exosomes will further encourage the presence of growth factors necessary for axon growth, myelination, and nerve reinnervation. Difficulties arise when attempting this treatment method. Effective treatment requires accurate mapping of the mechanisms that guide cellular differentiation and release neurotrophic support.

Before exosomal therapeutics can become an effective clinical option, limitations such as upscale, site specificity, storage, and quality assurance must be addressed. Upscaling cellular expansion for EV production has been shown to affect cell quality and, in turn, the cargo and concentration loaded within exosomes. A practical and repeatable upscaling technique that ensures minimal difference in cell and exosome quality from static to large-scale dynamic cultures has yet to be devised. After upscale, long-term storage must also be addressed. It is pivotal to store EVs for long periods in an economically feasible way to ensure clinical viability. Research is inconclusive, and many different methods such as lyophilization, storage buffer, and storage temperature still need to be studied. Despite having some site specificity due to the markers on their surface, EVs often accumulate in unintended sites like the liver or spleen. Current research focuses on increasing site specificity via exosomal surface modifications and cargo. The results are promising, but considering the effects of culture conditions on EV formation, they should be tackled along with engineered modifications to produce more site-specific exosomes. More research on engineered EV surface modifications, storage, particle reconstitution, clearance within the body, and long-term outcomes are all avenues of research that are of interest in cell-free therapy. Furthermore, a method of EV isolation to maximize exosome production as well as the optimal combination of materials necessary to enhance the regenerative microenvironment must be universally established. With EVs becoming more and more prevalent in research, it is critical to note that many safety barriers must be addressed. Regarding MSCs, there are conflicting data regarding safety. While the research suggests human MSC EVs are nontoxic, these studies are in vitro cultures with small dosages relative to those needed in clinical work [305]. Despite being nontoxic, there is conflicting evidence regarding oncogenesis derived from MSC EVs. While EVs cannot grow tumors, they can inhibit or upregulate tumor growth and metastasis [306,307,308]. In a 2021 study by Tan et al., they proposed that these conflicting data could be due to the heterogeneity of the MSC source, EV isolation methodology, or tumor model utilized [309]. Nonetheless, more must be known about EVs regarding oncogenesis and other safety concerns prior to clinical applications.

## Figures and Tables

**Table 1:** Various types of diabetic neuropathy categorized into five major categories.

Form of DPN	Description	Reference
Focal	Affecting 1 or a singular group of nerves (i.e., carpal tunnel).	[39]
Multifocal Peripheral Neuropathy	Length-dependent motor/sensory neuropathy.	[24]
Autonomic	Loss of involuntary bodily function.	[40]
Diabetic Amyotrophy (Proximal Neuropathy)	Unilateral or bilateral pain and sensory loss and muscular atrophy in quadriceps, hips, and gluteus maximus.	[24]
Idiopathic Neuropathy	Undetermined etiology of neuropathy.	[41]

**Table 2:** Description of the five types of drugs used when treating cancerous agents.

Drug	Treated Condition	CIPN Pathogenesis
Platinum Compounds	Tumors in cranium, digestive, urinary, respiratory, and reproductive systems.	Mitochondrial dysfunction. Increased oxidative stress. Voltage-gated K^+^ and Na^+^ hyperactivity.
Taxanes	Tumors in breast, ovaries, prostate, lungs, and bladder.	Mitochondrial dysfunction. Increased oxidative stress. Voltage-gated K^+^ and Na^+^ hyperactivity. Altered functionality of skin-based receptors (Aβ, C, and Aδ nerve fibers).
Vinca Alkaloids	Tumors in kidneys, liver, lungs, breast, and brain. Hematological malignancies, testicular, and non-small cell lung cancer.	Mitochondrial dysfunction. Microtubule function inhibition.
Immunomodulators	Example: thalidomide. MM, glioblastoma, breast, and prostate cancer.	Inhibition of growth factors (VEGF, TNF-α, NF-kB, b-FGF). ROS activation. Induced hypoxia and ischemia.
Proteosome Inhibitors	Example: bortezomib. Progressive, relapsed, or refractory MM.	Mitochondrial dysfunction. Increased oxidative stress. Increased apoptosis via release of Ca^2+^ in endoplasmic reticulum.

**Table 3:** Beneficial properties of each protein within the ECM.

Protein	Properties	Benefit to Neural Regeneration	Reference
Elastin	Highly elastic, water-soluble, hydrophobic.	Promotes cellular adhesion, proliferation, stem cell differentiation, the release of growth factors, drug delivery.	[169]
Fibrinogen	Produces fibrin network, composed of polypeptide chains.	Facilitate stem cell proliferation, adhesion, and differentiation.	[170]
Laminin	Abundant in native ECM.	Basement membrane. Facilitate cellular attachment, differentiation, and neurite outgrowth.	[171,172]
Silk	Naturally occurring in ECM.	Promotes oxygen and permeability. Biodegradable. Supports SC and neuron growth and attachment.	[173,174]
Collagen	Abundant in native ECM.	Fibroblast proliferation, angiogenesis, regulation of pro- and anti-inflammatory response.	[175,176]
Hyaluronic Acid	Abundant in embryonic tissue and ECM.	Maintains ECM, regulates binding proteins in cellular adhesion, proliferation, pro/anti-inflammatory response depending on molecular weight.	[177]

**Table 4:** Studies that utilized MSCs in neuropathic treatment.

MSC Source	Neuropathy Treated	Title of Study	Reference
hUC-MSC	DPN	Human umbilical cord-derived mesenchymal stem cells prevent the progression of early diabetic nephropathy through inhibiting inflammation and fibrosis.	[216]
BM-MSC	DPN	The bone marrow-derived mesenchymal stem cells (BMSCs) alleviate diabetic peripheral neuropathy induced by STZ via activating GSK-3β/β-catenin signaling pathway.	[217]
ASC	DPN	Treatment with adipose tissue-derived mesenchymal stem cells exerts anti-diabetic effects, improves long-term complications, and attenuates inflammation in type 2 diabetic rats.	[218]
hMSC	CIPN	Nasal administration of mesenchymal stem cells prevents accelerated age-related tauopathy after chemotherapy in mice.	[219]
BM-MSC	CIPN	Bone marrow-derived mesenchymal stem cells alleviate paclitaxel-induced mechanical allodynia in rats.	[220]
MSC	CIPN	Nasal administration of mesenchymal stem cells reverses chemotherapy-induced peripheral neuropathy in mice.	[221]
ASC	CIPN	Adipose-derived stem cells decrease pain in rat model of oxaliplatin-induced neuropathy: Role of VEGF-A modulation.	[222]
hASC and hUC-MSC	Neuropathic symptoms via partial sciatic nerve ligation	Intravenous administration of human mesenchymal stem cells derived from adipose tissue and umbilical cord improves NP via suppression of neuronal damage and anti-inflammatory actions in rats.	[223]
ASC	Peripheral nerve injury repair for NP relief	Role of adipose tissue grafting and adipose-derived stem cells in peripheral nerve surgery.	[194]

**Table 5:** Studies that utilized exosomes in neuropathy treatment.

Exosome Source	Neuropathy Treated	Title of Study	Reference
hMSC	DPN	Treatment of diabetic peripheral neuropathy with engineered mesenchymal stromal cell-derived exosomes enriched with microRNA-146a provide amplified therapeutic efficacy.	[237]
hBM-MSC	DPN	Exosomes derived from atorvastatin-pretreated MSC accelerate diabetic wound repair by enhancing angiogenesis via AKT/eNOS pathway.	[240]
hBM-MSC	DPN	Melatonin-stimulated MSC-derived exosomes improve diabetic wound healing through regulating macrophage M1 and M2 polarization by targeting the PTEN/AKT pathway.	[241]
SC-EV	DPN	Exosomes derived from Schwann cells ameliorate peripheral neuropathy in type 2 diabetic mice.	[247]
CEC-sEV	CIPN	Small extracellular vesicles ameliorate peripheral neuropathy and enhance chemotherapy of oxaliplatin on ovarian cancer.	[238]
hBM-MSC-EVs	CIPN	Bone marrow mesenchymal stem cells and their derived exosomes resole doxorubicin-induced chemobrain: Critical role of their miRNA cargo.	[248]
hUC-MSC	Microglial activation of NP	Huc-MSCs-derived exosomes attenuate NP by inhibiting activation of the TLR2/MyD88/NF-kB signaling pathway in the spinal microglia by targeting Rasad2.	[249]
MSC	Microglial activation of NP	Mesenchymal stem cell-derived extracellular vesicles carrying miR-99b-3p restrain microglial activation and NP by stimulating autophagy.	[250]
BM-MSC	NP via sciatic nerve chronic constriction injury	Exosomes carried miR-181c-5p alleviates NP in CCI rat models.	[251]
MSC	NP via spinal neuroinflammation	Extracellular vesicles derived from mesenchymal stem cells alleviate neuroinflammation and mechanical allodynia in interstitial cystitis rats by inhibiting NLRP3 inflammasome activation.	[252]
hUC-MSC	Alleviate inflammatory pain	Huc-MSCs-derived exosomes attenuate inflammatory pain by regulating microglia pyroptosis and autophagy via the miR-146a-5p/TRAF6 axis.	[253]

## Abbreviations

AC	Alternating current	NAD^+^	Oxidative nicotinamide adenine dinucleotide
hASC	Human adipose derived MSC	NADH	Reductive nicotinamide adenine dinucleotide
AKT	Protein kinase B	NAM	Nicotinamide
ATP	Adenosine triphosphate	NEFL	Neurofilament light polypeptide
Aβ	Alpha beta	NEFM	Neurofilament medium polypeptide
Aδ	Alpha delta	NESTIN	Neuroepithelial stem cell protein
BBB	Blood–brain barrier	NK-kB	Nuclear factor-kappa beta
BDNF	Brain-derived neurotrophic factor	NGF	Nerve growth factor
b-FGF	Fibroblast growth factor	nm	Nanometers
hBM-MSC	Human bone marrow derived MSC	NMDA	N-methyl-D-aspartate
Ca^2+^	Calcium	NMN	Nicotinamide mononucleotide
CD44	Cluster of differentiation 44	NMNAT	Nicotinamide mononucleotide adenylyltransferase
CHMP4C	Chromatin modified protein 4C	NP	Neuropathic pain
CIPN	Chemotherapy-induced peripheral neuropathy	NR	Nicotinamide riboside
CNS	Central nervous system	nSMase2	Neutral sphingomyelinase 2
CT	Computerized tomography		
DC	Direct current	NT-3	Neurotrophin-3
DHT	Dihydrotestosterone	NURR1	Dopaminergic neuron marker
DPN	Diabetic peripheral neuropathy	OCT-3/4	Octamer transcription factor 3 and 4
DSN	Distal symmetric neuropathy	P0	Protein zero
ECM	Extracellular matrix	PI3K	Phosphatidylinositol 3-kinase
ER	Endoplasmic reticulum	PAX6	Paired box 6
ESCs	Embryonic stem cells	PC	Phosphatidylcholine
ESCRT	Endosomal sorting complex required for transport	PCL	Polycaprolactone
EVs	Extracellular vesicles	PE	Phosphatidylethanolamine
FasL	Fas ligand	PGA	Poly-glycolic acid
FBS	Fetal bovine serum	PI	Phosphatidylinositol
FDA	U.S. Food and Drug Administration	PLA	Polylactic acid
FK506	Tacrolimus	PLGA	Poly-dl-lactic-co-glycolic acid
GABA	Gamma-aminobutyric acid	PMP22	Peripheral myelin protein-22
GAP-43	Growth-associated protein 43	PN	Peripheral neuropathy
GDF3	Growth and differentiation factor 3	PNS	Peripheral nervous system
GDNF	Glial cell-derived neurotrophic factor	PS	Phosphatidylserine
GFAP	Glial fibrillary acidic protein	PSA	Pressure sensitive adhesive tape
GluN1	Glycine-binding subunits	PVA	Polyvinyl acetate
GM	Gangliosides	qPCR	Quantitative polymerase chain reaction
GPCR	G protein-coupled receptor		
GPI	Glycosylphosphatidylinositol	RA	Retinoic acid
GTPase	Guanosine triphosphate	Rab GTPases	Ras-associated binding guanosine triphosphates
HA	Hyaluronic acid	REX1	Reduced expression 1
hASC	Human ASC	ROS	Reactive oxygen species
hBM-MSC	Human BM-MSC	rSCs	Repair Schwann cells
Hrs	Hepatocyte growth factor-regulated tyrosine kinase substrate	RT-PCR	Reverse transcription polymerase chain reaction
HF	High frequency	S100B	Calcium-binding protein B
HLA-DR	Human leukocyte antigen	SARM1	Sterile alpha and toll/interleukin-1 receptor motif-containing 1
HPL	Human platelet lysate	SC	Schwann cell
Hsc	Heat shock cognate	SHH	Sonic Hedgehog protein
Hsp	Heat shock protein	SMPD2	Sphingomyelin phosphodiesterase 2
hUC	Human umbilical cord	SOX2	Sex determining region Y-box 2
Hz	Hertz	STAM1	Signal transducing adaptor molecule
IGF-1	Insulin-like growth factor 1	STZ	Streptozotocin
IL	Interleukin	TENS	Transcutaneous electrical nerve stimulation
ILV	Intraluminal vesicles	TfR	Transferrin receptor
iPSCs	Induced pluripotent stem cells	TGF-β1	Transforming growth factor-beta 1
JNK	C-Jun N-terminal kinase	TH	Tyrosine hydroxylase
K^+^	Potassium	TLR4	Toll-like receptor member 4
KLF4	Kruppel-like factor 4	TNF-α	Tumor necrosis factor
kPa	Kilopascal		
LAMP1	Lysosomal-associated membrane protein 1	Trp	Tryptophan
LF	Low frequency	TRAIL	TNF related apoptosis-inducing ligand
mA	Milliamps	TSG	Tumor susceptibility gene
MAL	Myelin and lymphocyte protein	Tuj1	Class III beta-tubulin
MAPK	Mitogen-activated protein kinase	hUM-MSC	Human umbilical cord derived MSC
MEK/ERK	Kinase extracellular signaling regulation pathway	VEGF	Vascular endothelial growth factor
MHC	Major histocompatibility complex	VPS	Vascular protein sorting-associated protein
miRNA	MicroRNA	VTA1	Vacuolar protein sorting-associated protein
MITF	Melanocyte including transcription factor	WD	Wallerian degeneration
MM	Multiple myeloma	WHO	World Health Organization
MRI	Magnetic resonance imaging	Wlds	Wallerian degeneration slow
mRNA	Messenger RNA	μs	Microseconds
MSCs	Mesenchymal stromal cells	µm	Micrometers
MVB	Multivesicular bodies	2D	Two-dimensional
NA	Nicotinic acid	3D	Three-dimensional
Na^+^	Sodium		
